# Response of Near-Inertial Shear to Wind Stress Curl and Sea Level

**DOI:** 10.1038/s41598-019-56822-z

**Published:** 2019-12-31

**Authors:** Jing Gao, Jianing Wang, Fan Wang

**Affiliations:** 1 Key Laboratory of Ocean Circulation and Waves, Institute of Oceanology, Center for Ocean Mega-Science, Chinese Academy of Sciences, Qingdao, 266071 China; 2 University of Chinese Academy of Sciences, Beijing, China; 3 Laboratory for Ocean and Climate Dynamics, Qingdao National Laboratory for Marine Science and Technology, Qingdao, China

**Keywords:** Hydrology, Physical oceanography

## Abstract

Near-inertial waves (NIWs) contain a pronounced portion of shear energy in the internal wave field and is of great importance to deep ocean mixing. However, accurate simulation of NIWs remains a challenge. Here we analyzed 3-year long mooring observation of velocity profiles over 80–800 m to study the responses of near-inertial downward shear to varying wind stress curls and sea level anomalies (SLAs). It is demonstrated that moderate (even weak) cyclone makes more contributions to enhanced shear below the pycnocline than very strong cyclone. Because very strong curl can stall the downward propagation of large shear. The large positive and negative SLAs cause the accumulation of large shear in the lower and upper parts of the pycnocline through inducing downwelling and upwelling motions, respectively. Time variation of near-inertial shear was strongly influenced by cases of large curls and interannual variation of SLA, and thus did not follow the seasonal variation of wind stress. Our analyses suggest that matched fields of wind stress curl and SLA, and well representing the ocean response to moderate cyclone are needed in simulating the role of NIWs on mixing.

## Introduction

**Figure 1:**
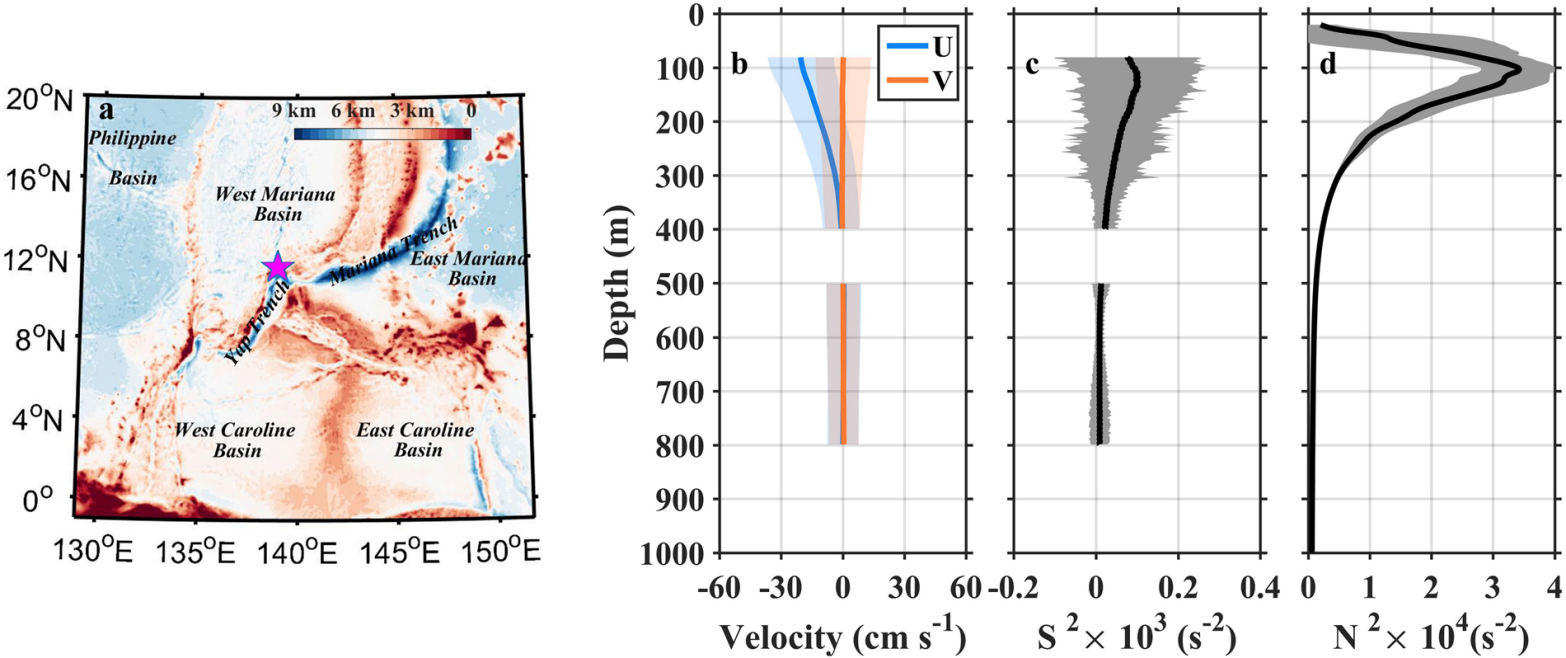
Mean velocity and squared shear from observation, and squared buoyancy frequency from EN4.1 during the mooring measurement period. **(a)** Topography of the western Pacific Ocean with the mooring location (pink star) at the center. Vertical profiles of mean (thick lines) and standard deviation (shaded areas) of **(b)** zonal (blue, *U*) and meridional (orange, *V*) velocities, **(c)** squared shear (*S*^2^), and **(d)** square buoyancy frequency (*N*^2^). In **(d)**, *N*^2^ is calculated from EN4.1 monthly temperature and salinity. Figures are plotted using MATLAB R2015b (http://www.mathworks.com/).

Near-inertial waves (NIWs) form a prominent peak in the frequency spectrum of ocean current and contain a pronounced portion of shear energy in the internal wave field^1^. The past decades have been seen significant progress in understanding the physics of NIWs (e.g., a recent review by Alford *et al*.^2^). Wind-induced NIWs are primarily triggered by the wind work on near-inertial oscillation in the mixed layer, and can propagate vertically into the pycnocline and deep ocean. Eventually, breaking of these waves has been believed to provide one-half energy for diapycnal mixing required to sustain the meridional overturning circulation^3–6^. However, recent model results^5,7,8^ show that the role of wind-induced near-inertial energy might have been largely overestimated. The question about how much of the near-inertial energy input by wind is actually available for the deep ocean mixing is still being debated^8^. Thus, the need to better understand the driving and influencing mechanisms for vertical propagation of NIWs looms large. In this study, we will address this issue by examining 3-year long mooring observation of near-inertial velocity shear in the upper ocean in the Western Pacific (Fig. 1a). It is shear rather than velocity that destabilizes flow, transfers kinetic energy from the mean to the turbulent fields, and finally drives mixing in the open ocean. Therefore, knowledge of distribution and variability of velocity shear is an important goal of physical oceanography community^9^.

**Figure 2:**
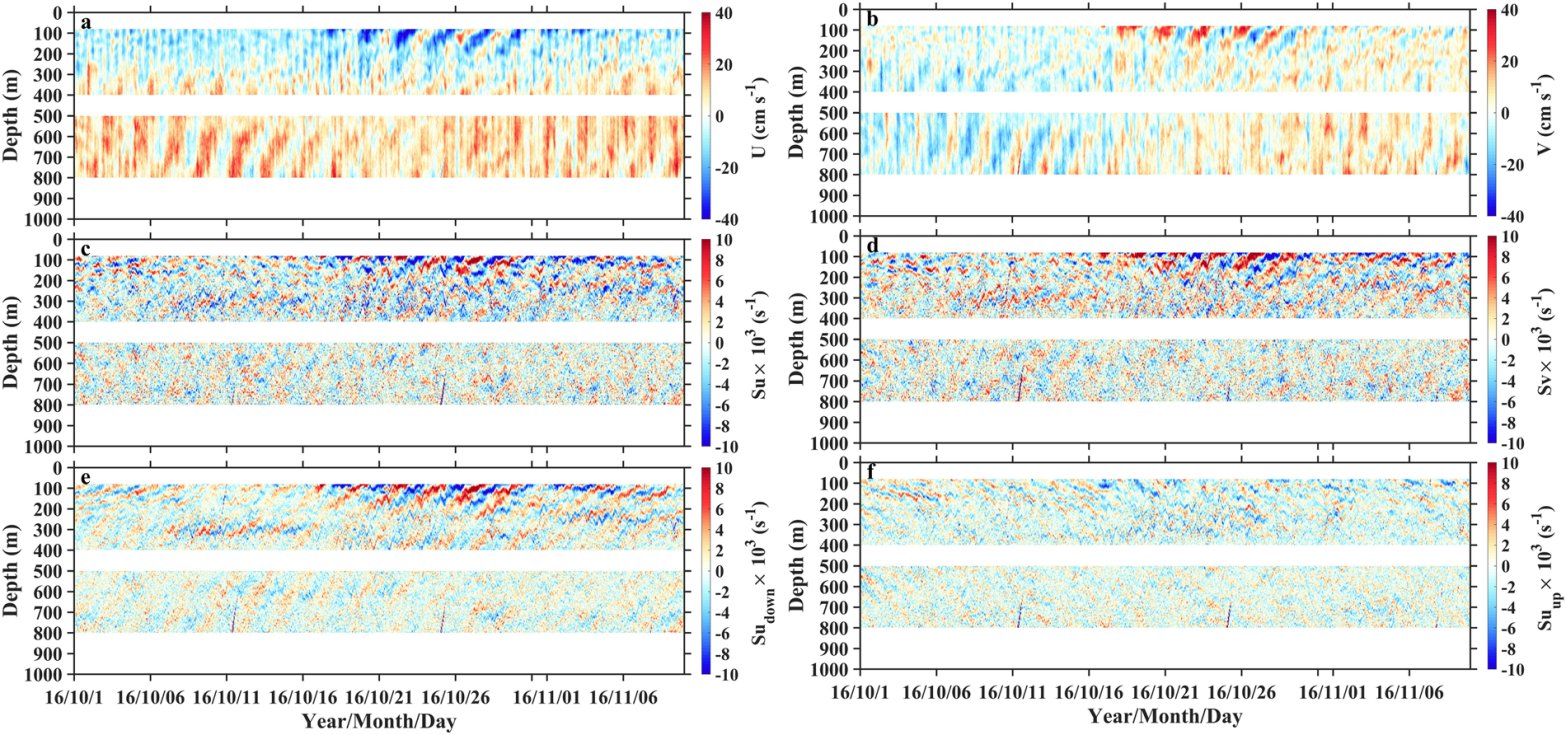
Observed velocities, total shears, and their upward and downward energy propagating components. Time-depth variations of **(a)** zonal and **(b)** meridional velocities (*U* and *V*), **(c)** zonal and **(d)** meridional total shears (*Su* and *Sv*), and **(e)** downward and **(f)** upward energy propagating components (*Su*_*down*_ and *Su*_*up*_) of *Su* from 1 October 2016 to 10 November 2016. Separation of **(e)** and **(f)** are accomplished via 2D Fourier filtering (Methods). Figures are plotted using MATLAB R2015b (http://www.mathworks.com/).

**Figure 3:**
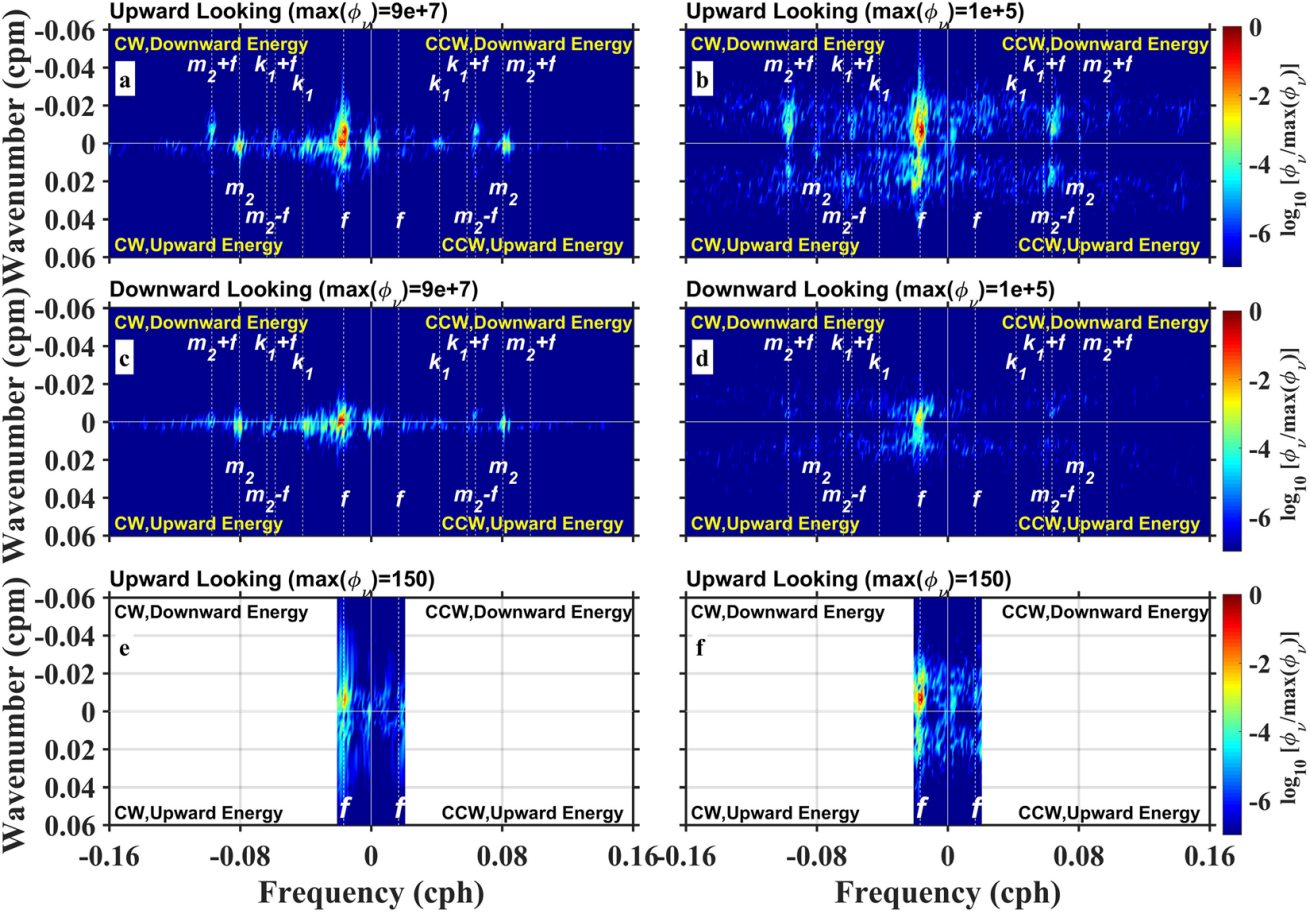
Wavenumber-frequency spectra of WKB-scaled rotary velocities and shears. Spectra in **(a)** and **(c)** are derived from hourly velocities observed by one upward-looking and one downward-looking ADCPs, spanning over 80–400 m and 500–800 m, respectively. Panels **(b)** and **(d)** are the same as panels **(a)** and **(c)**, but for hourly shears. Panels **(e)** and **(f)** are the same as panel **(b)**, but for daily shear from HYCOM and mooring observation, respectively. In all spectra, the period of data is from 1 October 2016 to 10 November 2016, *f*, *m*_2_, and *k*_1_ denote inertial, semi-diurnal, and diurnal frequencies, and CW and CCW represent clockwise and counter-clockwise rotations. Figures are plotted using MATLAB R2015b (http://www.mathworks.com/).

Two ADCPs on subsurface mooring observed the strong westward flowing North Equatorial Current with velocity larger than 20 cm s^−1^ from 80 m to 200 m^10,11^, corresponding to the pycnocline layer with *N*^2^ larger than 2 × 10^−4^ s^−2^, and east-west oscillating flow from 200 m to 800 m, corresponding to the sub-pycnocline layer with *N*^2^ smaller than 2 × 10^−4^ s^−2^ (Fig. 1b,d). The mean shear energy was intensified in the pycnocline and became weak below 500 m, and vertical distribution of shear energy resembled that of squared buoyancy (Fig. 1c,d). Both the zonal and meridional velocity fields were dominated by the near-inertial internal waves with a period of 59.5 h (Fig. 2a,b). The phase lines slant upward with time, evidently captured by eyes. This indicates upward phase propagation and downward energy propagation (Fig. 2a–d). Time-depth variations of vertical shears resembled those of velocities but with higher vertical wavenumber and more clearly coherent features of upward and downward energy propagations. The zonal shear field was then decomposed into the components with downward and upward energy propagations (Methods, Fig. 2e,f). The downward shear (energy propagation, same hereinafter) intensity was much larger than upward shear. These characteristics can be further quantified in the wavenumber-frequency spectra (Methods, Fig. 3). Both velocity and shear spectra show a primary peak at the negative near-inertial frequency (-*f*), indicating the dominance of clockwise rotation of near-inertial motion in the northern hemisphere. Furthermore, the near-inertial peaks appeared in both positive and negative wavenumber domains, suggesting the co-occurrence of upward and downward energy propagations, respectively. In comparing with near-inertial energy in negative and positive wavenumbers and in different depths observed by upward- and downward-looking ADCPs, the former situations were larger than the latter one. This suggests that downward propagation (energy propagation, same hereinafter) and shallower layers contained more near-inertial energy than upward propagation and deeper layers, respectively. In addition, the spectra also show peaks at semi-diurnal (*m*_2_) and diurnal (*k*_1_) tidal frequencies, and some subharmonic frequencies (e.g., *m*_2_ ± *f*, *k*_1_ + *f*). Frequencies of *m*_2_ and *k*_1_ in velocity spectra (Fig. 3a,c) have larger energy than that in the shear spectra (Fig. 3b,d), indicating that semi-diurnal and diurnal tidal energy are dominated by low-mode motions. The appearances of *m*_2_ ± *f* and *k*_1_ + *f* peaks can be attributed to nonlinear wave-wave interaction^12–16^ or vertical heaving of the near-inertial shear by tidal motions^17–19^. We also evaluate the model capability of simulating the NIWs. The wavenumber-frequency spectra of daily shear obtained from Hybrid Coordinate Ocean Model (HYCOM) output and our observation at the mooring site are compared (Fig. 3e,f). The maximum of the downward shear spectral energy from the model is 72% lower than that from observation, suggesting the model cannot well reproduce the downward propagation of NIWs. This encourages us to do further studies on driving and influencing mechanisms of near-inertial shear energy.

**Figure 4:**
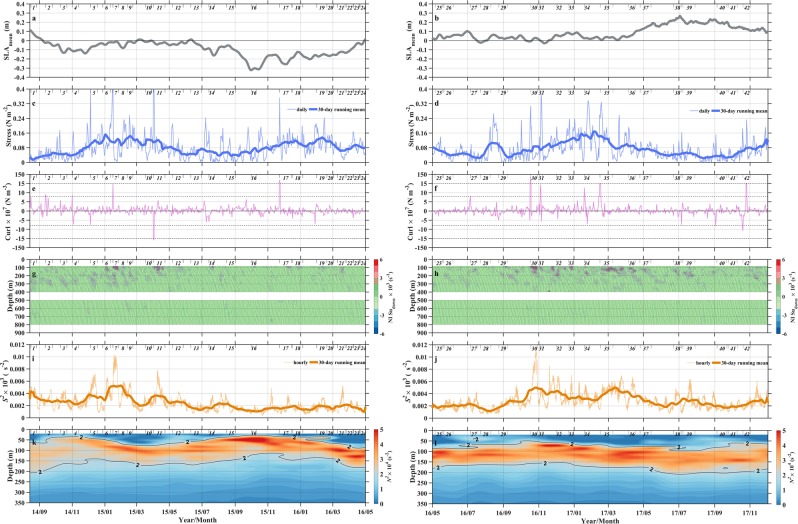
Time series of (**a**,**b**) SLA, (**c**,**d**) wind stress and (**e**,**f**) its curl, (**g**,**h**) near-inertial downward shear profiles, (**i**,**j**) mean shear energy over 80–800 m, and (**k**,**l**) squared buoyancy frequency profiles. In (**a–f**,**k–l**), data are obtained by taking an average within 138.6–139.6°E and 11.1–12.1°N. In (**c-d**,**i-j**), the thin and thick lines denote original values and their 30-day running mean. In **(e**,**f)**, y-axis is non-uniform and horizontal dashed lines at −15 × 10^−7^, −8 × 10^−7^, 8 × 10^−7^, and 15 × 10^−7^ N m^−3^ server as a guide to separate strong, moderate, and weak curls. In **(k,l)**, contour of 2 × 10^−4^ s^−2^ indicates the location of pycnocline. Left and right panels correspond to the negative and positive SLAs, respectively. In (**a**–**l**), 42 segments are selected for the following case study, and are denoted by two short vertical lines with a number at center in the top part of each panel. Figures are plotted using MATLAB R2015b (http://www.mathworks.com/).

The intensity of observed downward shear displayed strong variation with depth and time (Fig. 4g,h). According to previous studies^2,9^, the local shear is dominated by slow-moving high modes NIWs, while low modes NIWs move quickly and their energy is presumed to dissipate far from the generation site, though the exact fate is unknown. Thus, we ignore the effect of remote-generated NIWs on observed shear in this study. The downward shear energy generally decreased with increasing depth, occasionally some large value appearing at the lower layer. Previous studies^20,21^ suggest that the time-depth variation of shear energy can be influenced by variability of sea level anomaly denoting eddy chimney effect (Fig. 4a,b), wind stress (Fig. 4c,d) and its curl (Fig. 4e,f), and ocean mixed layer depth (Fig. 4k,l). Interestingly, our observation period can be nearly equally separated into two parts according to the SLA. The former (rest) period from August 2014 to April 2016 (from May 2016 to November 2017) corresponds to the negative (positive) SLA, denoting the positive (negative) relative vorticity of the mesoscale flow. The variation of local wind stress was broadly seasonal with high-frequency oscillation superimposed, and its curl was dominated by high-frequency variability. The monthly variation of pycnocline depth obtained from EN 4.1 *N*^2^ cannot show the response to high-frequency variability of wind stress curl, but was negatively correlated with low-frequency variability of SLA. The shallowing pycnocline led to thinner mixed layer depth when the SLA was negative, and vice versa^22^ (Fig. 4k,l).

**Figure 5:**
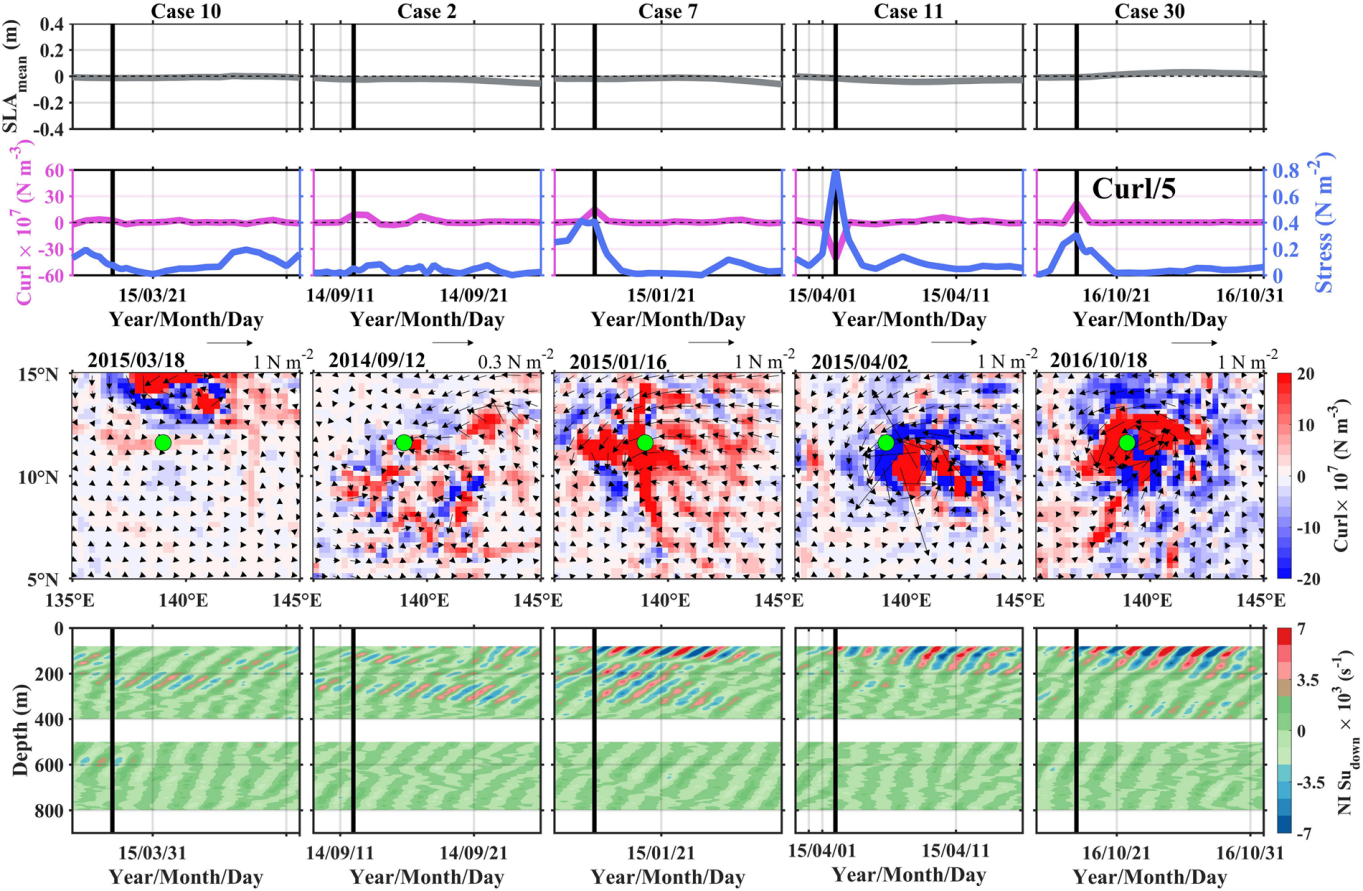
Scenario 1: Evaluating responses of near-inertial downward shear to increasing wind stress curls (from left to right). Time series of SLA (1^st^ row), original wind stress (blue) and its curl (purple, 2^nd^ row), and near-inertial downward shear profiles (4^th^ row) during (from left to right) Cases 10, 2, 7, 11, and 30. For the 3^rd^ row, horizontal fields of wind stress (vectors) and its curl (color shading) on the forcing start day are shown (denoted by black lines in the other three rows), and the green dots denote the mooring location. In Case 30, the curl in 2^nd^ row is scaled by a factor of 5 to fit on the same axes. Figures are plotted using MATLAB R2015b (http://www.mathworks.com/).

Next, we will discuss the influences of wind stress curl and SLA on the near-inertial downward shear by analyzing two scenarios. Compared to wind stress, its curl could trigger the strong downward shear more efficiently through pumping the base of the mixed layer^2,9^. In addition, wind stress and its curl are usually correlated on a daily time scale. Thus, we only focus on wind stress curl here. Scenario 1 is designed to examine the response of near-inertial downward shear to varying intensities of wind stress curl. Five cases with similar SLA and increasing magnitudes of wind stress curl (from left to right columns in Fig. 5) are contained in this Scenario. With curl intensity increasing from Case 10 to Case 7, the triggered downward shear strengthens, and strong shear with absolute value larger than 4 × 10^−3^ s^−1^ can extend to 350 m. With curl intensity further increasing from Case 11 to Case 30, the triggered downward shear further strengthens, but strong shear with the same intensity is confined to the depth above 200 m. This suggests that the wind stress curl has a two-fold impact on the downward shear. The increasing curl, on the one hand, triggers enhanced downward shear through pumping the base of the mixed layer^2,4,23^, and on the other hand, puts constraint on the downward propagation of shear.

The two-fold processes are associated with the change of stratification profile during the strong curl passing the mooring site. According to previous observational studies^24,25^, the strong positive wind stress curl can cause shallower pycnocline, and increase in *N*^2^ in the pycnocline and decreases in *N*^2^ below the pycnocline through inducing upwelling motion. Decrease in *N*^2^ below the pycnocline leads to smaller vertical internal wave flux (*F*_*E*_), meaning the confining of downward shear^26–28^. This can be understood as follows. *F*_*E*_ can be obtained by calculating internal wave–induced velocity–pressure perturbations correlation or by multiplying vertical group velocity (*C*_*gz*_) and energy density (Methods). First, internal wave–induced pressure perturbation is positively correlated with *N*^26^, and thus decreasing *N*^2^ below the pycnocline corresponds to smaller *F*_*E*_. Second, based on the definition of *C*_*gz*_, decreasing *N*^2^ leads to smaller *C*_*gz*_ and resultant *F*_*E*_. The variation of *C*_*gz*_ can be seen from Fig. 5. With increasing curl from Case 7 to Case 30, the slant angle of shear phase with respect to vertical direction is reduced, corresponding to decreasing *C*_*gz*_. Furthermore, shallower pycnocline and enhanced *N*^2^ within it induced by strong curl are combined to make the intensified shear only accumulating in the shallower depth. This scenario may imply that it is the moderate intensity of wind stress curl that can make more contributions to the deep ocean mixing.

**Figure 6:**
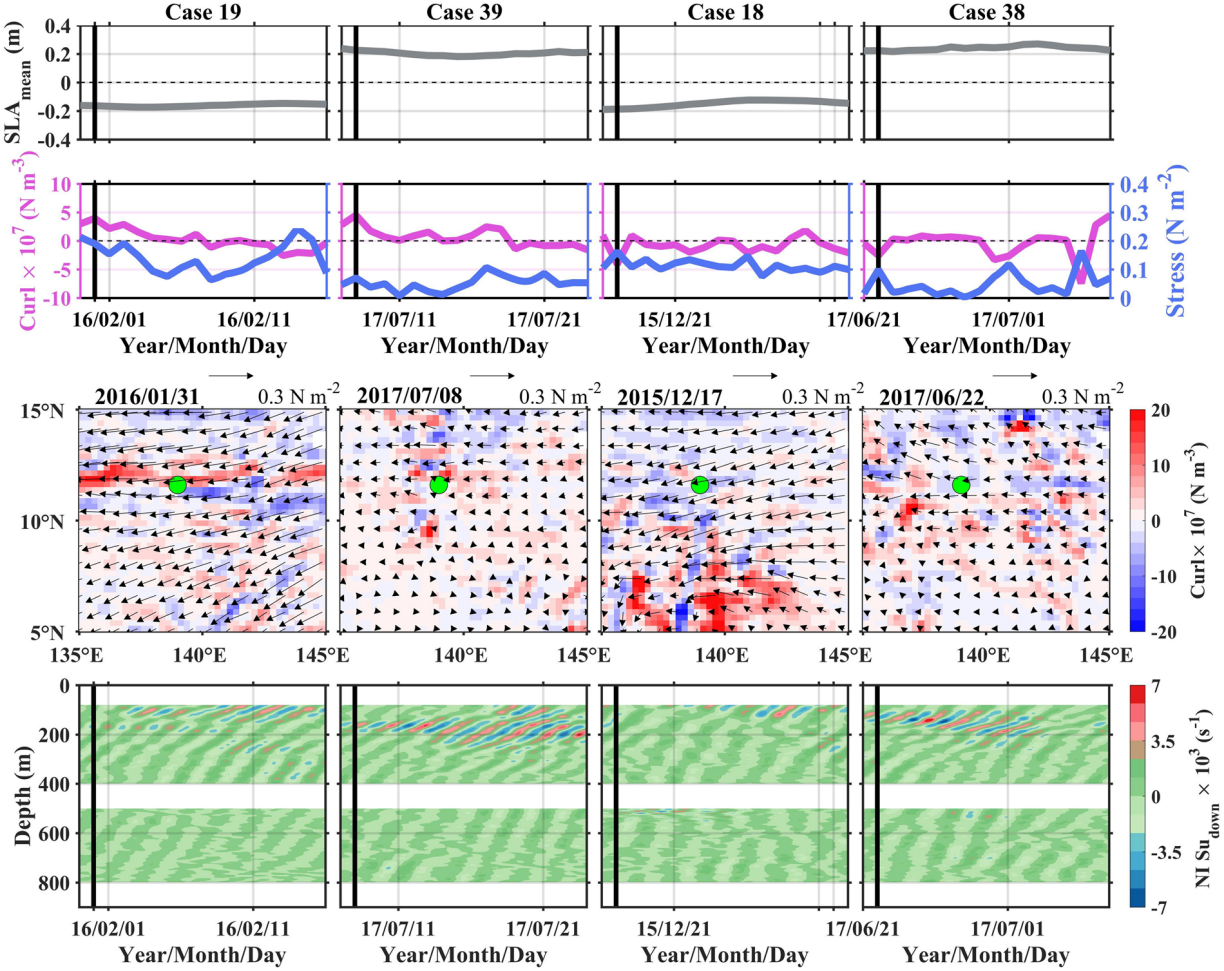
Scenario 2: Examining responses of near-inertial downward shear to large positive and negative SLAs. Same as Fig. 5 but for Cases 19, 39, 18, and 38. Note that the legend for the wind stress vector is different from that in Fig. 5. Figures are plotted using MATLAB R2015b (http://www.mathworks.com/).

Scenario 2 is constructed to evaluate the impact of SLA on time-depth variation of downward shear (Fig. 6). Four cases are contained in this scenario. The first two cases are under comparable forcing wind stress curls, but with inverse SLAs. The latter two cases resemble the first two, but their curls are negative. The difference of downward shear between the positive and negative SLA cases is striking. For the negative SLA cases (Cases 19 and 18), the strong shear with absolute value larger than 4 × 10^−3^ s^−1^ is confined at the depth above 120 m. For the positive SLA case (Cases 39 and 38), the shear with similar intensity can extend into the depth of 220 m. The positive SLA usually corresponds to downwelling motion, leading to increases in the depths of the base of the mixed layer and pycnocline, and the pycnocline thickness^20–22,28^. The deepening of the base of the mixed layer means the generation depth of downward shear deeper. Furthermore, the deepening and thickening of pycnocline facilitate the downward propagation flux of internal waves. The situation is reversed during the period of negative SLA. The negative SLA corresponds to shallower generation depth and smaller vertical flux of internal waves.

The above studies revealed that the intensity and propagation depth of enhanced shear are related to wind stress curl and SLA. However, these results are only presented as the “scenario” style, and still needs supports from significant statistics. We next do the scatter and ensemble mean analyses using all available wind-induced NIW cases. There is a total of 42 cases, the detailed information of which are given in Table 1. Each case contains 121–529 hourly shear profiles and corresponds to one forcing curl and one influencing SLA on the start day. Persisting periods of triggered shear during 42 cases add up to 587 days, accounting for approximate 50% of the whole measurement period. Note that Case 11 is strongly influenced by the fast-moving strong cyclone (Fig. 5), although its forcing curl is negative due to insufficient time-space resolution, and we thus set the curl to its absolute value. We also try to use 6-hourly wind data (e.g., Cross-Calibrated Multi-Platform (CCMP) gridded surface vector winds) that with the same spatial resolution (0.25°) as ASCAT. Their results show that strong wind stress curls are underestimated as compared to those from ASCAT, although there is improvement in time resolution (figure not shown).

**Table 1:** Start day, forcing wind stress curl, influencing SLA, persisting period of triggered shear, and number of hourly shear profiles for all 42 wind-induced NIW cases.

Serial number	Start day (Year-Month-Day)	Forcing wind stress curl × 10^7^ (N m^−3^)	Influencing SLA (m)	Persisting period of triggered shear (day)	Number of triggered hourly shear profiles
1	2014-08-16	7.7309	0.1063	10	241
2	2014-09-12	8.9439	−0.0245	13	313
3	2014-10-10	5.8852	−0.0885	12	289
4	2014-11-01	9.0258	−0.1107	12	289
5	2014-12-05	−7.5691	−0.079	13	313
6	2014-12-28	3.0088	−0.0326	14	337
7	2015-01-16	14.8539	−0.0199	10	241
8	2015-01-26	2.3569	−0.0249	17	409
9	2015-02-12	−0.2764	−0.0189	10	241
10	2015-03-18	2.2459	−0.0138	11	265
11	2015-04-02	−41.0159* (41.0159)	−0.0153	21	505
12	2015-05-07	3.4445	−0.0407	20	481
13	2015-06-10	1.6331	0.011	20	481
14	2015-07-14	−5.9552	−0.087	10	241
15	2015-08-16	1.2309	−0.0591	17	409
16	2015-10-01	1.7478	−0.3225	9	217
17	2015-11-23	67.649	−0.2001	22	529
18	2015-12-17	−4.0933	−0.1873	19	457
19	2016-01-31	3.9408	−0.1635	19	457
20	2016-02-19	1.5402	−0.1592	13	313
21	2016-03-10	1.7129	−0.1454	14	337
22	2016-03-26	−0.1079	−0.1337	13	313
23	2016-04-09	−2.3643	−0.0745	11	265
24	2016-04-21	−1.0927	−0.0356	12	289
25	2016-05-06	−0.8534	0.0153	13	313
26	2016-05-21	1.2044	0.0225	16	385
27	2016-07-06	8.16	0.0775	11	265
28	2016-07-23	−0.0418	−0.0225	18	433
29	2016-08-24	−5.0716	0.0287	15	361
30	2016-10-18	110.1151	−0.0083	12	289
31	2016-11-04	14.3915	−0.002	5	121
32	2016-12-02	2.8402	0.0603	7	169
33	2016-12-23	3.1011	0.0817	8	193
34	2017-01-19	12.5951	0.0255	8	193
35	2017-02-15	18.1219	0.0152	22	529
36	2017-04-05	4.0527	0.0435	12	289
37	2017-04-25	1.9687	0.1137	19	457
38	2017-06-22	−2.5570	0.2227	13	313
39	2017-07-08	4.4939	0.2252	21	505
40	2017-09-03	−8.3613	0.223	17	409
41	2017-09-25	0.3186	0.1763	15	361
42	2017-10-20	−10.8836	0.1322	13	313

^*^Note that Case 11 is strongly influenced by the fast-moving strong cyclone (Fig. 5), although its forcing curl is negative due to insufficient time-space resolution, and we thus set the curl to its absolute value.

The forcing wind stress curl and influencing SLA are defined as their corresponding values on the start day.

**Figure 7:**
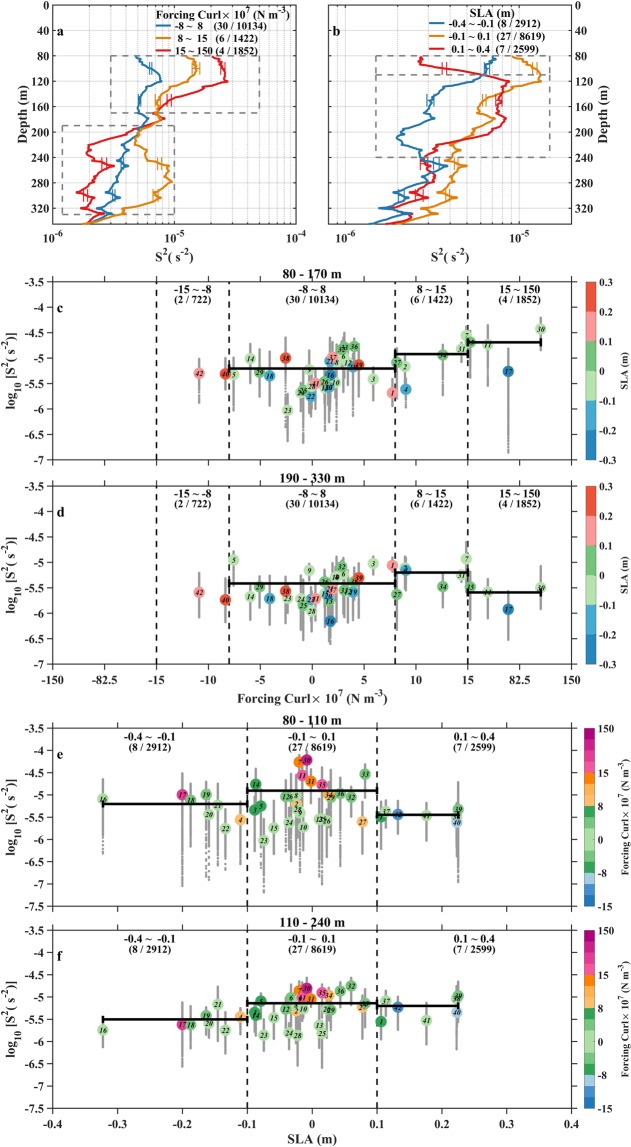
Ensemble and scatter plots of mean near-inertial downward shear energy (*S*^2^) with different wind stress curls and SLAs. Vertical distributions of hourly *S*^2^ averaged over ensembles of (**a**) weak, moderate, and strong wind stress curls and (**b**) negative, reference, and positive SLAs. For each ensemble, 95% confidence intervals derived from the bootstrap method are indicated by horizontal bars. Scatter diagrams of forcing curls versus case mean (color large dots) and hourly-profile mean (small gray dots) *S*^2^ over (**c**) 80–170 m and (**d**) 190–330 m (denoted by dashed box in panel a), with color depicting SLA. Scatter diagrams of SLA with case mean (color large dots) and hourly-profile mean (small gray dots) *S*^2^ over (**c**) 80–110 m and (**d**) 110–240 m (denoted by dashed box in panel b), with color depicting forcing curl. The ensemble ranges, the corresponding numbers of cases (number before slash in bracket) and hourly profiles (number after slash in bracket) are denoted in each panel. In (**c**–**f**), the number centered in large dots denotes the case serial number, vertical dashed lines serve as a guide to separate different ensembles, and three bold horizontal lines indicate the average value of case mean *S*^2^ over different ensembles. Figures are plotted using MATLAB R2015b (http://www.mathworks.com/).

Corresponding to the Scenario 1, we first separate all 42 cases into some typical ensembles of forcing wind stress curls. Forcing curls are divided into three ensembles, including the very strong ensemble with positive curl much larger than 15 × 10^−7^ N m^−3^, the moderate ensemble with positive curl over 8 × 10^−7^–15 × 10^−7^ N m^−3^, and the weak ensemble with curl over −8 × 10^−7^–8 × 10^−7^ N m^−3^. The strong and moderate negative curl ensembles are not involved in this study. Because there are only two negative curl cases with intensity larger than 8 × 10^−7^ N m^−3^ and we are uncertain that these two negative curls are caused by fast-moving cyclone due to insufficient time-space evolution or by the real anticyclone. The very strong, moderate, and weak ensembles contain 4 cases and 1852 shear profiles, 6 cases and 1422 shear profiles, and 30 cases and 10134 shear profiles, respectively. Figure 7a shows curl ensemble-mean of hourly shear energy profiles. At the depth above 170 m (upper dashed box in Fig. 7a), shear intensity is positively correlated with the level of forcing curl, and mean shear energy of the strong curl ensemble is larger than those of moderate and weak curl ensembles. At the depth over 190–330 m (lower dashed box in Fig. 7a), the mean shear energy of the moderate curl ensemble becomes most energetic, and is 4 times larger than that of strong curl ensemble. Such difference in shear energy may correspond to diapycnal diffusivity difference of one order of magnitude based on their relationships already established in other open ocean areas^29,30^. Based on 42 cases and their 14130 hourly shear profiles, we next do the scatter plots of forcing curl versus case mean (large color dots) and profile mean (small gray dots) shear energy in (80–170 m) and below (190–330 m) the pycnocline, with color depicting the corresponding SLA (Fig. 7c,d). At the depth over 80–170 m, there is an increasing trend of shear energy with increasing absolute value of forcing curl. The correlation can reach 0.76 if we exclude the cases which are strongly influenced by large positive and negative SLAs (dots with red and blue colors). In the weak curl range, Cases 6, 33, 36, and 37 appeared to be influenced by fast-moving large curl fields, which however cannot be fully resolved by daily wind data. Thus, their forcing curls tend to be smaller than the real values. At the depth over 190–330 m, almost all profile mean shear energy is reduced with the significant decrease in the strong curl range. The case mean and profile mean shear energy of very strong curls notably decrease by approximate 87%. This decreasing amplitude of strong curls are 40% and 49% higher than those of moderate and weak curls, respectively. The increasing trend of shear energy with forcing curl in the shallower depth is changed to the parabolic trend here, showing the peak shear energy in the moderate curl range.

Corresponding to the Scenario 2, we also first separate all 42 cases into some typical ensembles of SLAs. SLAs are divided into three ensembles, including the large positive ensemble with SLA between 0.1 and 0.4 m, the reference ensemble with SLA between −0.1 and 0.1 m, and the large negative ensemble with SLA between −0.4 and −0.1 m. The positive, reference, and the negative ensembles contain 7 cases and 2599 profiles, 27 cases and 8619 profiles, and 8 cases and 2912 profiles, respectively. For the ensembles of positive and negative SLAs (Fig. 7b), the difference of shear energy above 240 m is striking. At the depth above 110 m (upper dashed box in Fig. 7b), shear energy of the negative SLA ensemble is larger than that of the positive SLA ensemble. At the depth between 110 m and 240 m (lower dashed box in Fig. 7b)), the situation is reversed, and the mean shear energy of the positive SLA ensemble is 4 times larger than that of the negative SLA ensemble. Based on 42 cases and their 14130 shear profiles, we next do the scatter plots of SLA versus case mean (large color dots) and profile mean (small gray dots) shear energy over the upper (80–110 m) and lower (110–240 m) parts of pycnocline, with color depicting the corresponding forcing curl (Fig. 7e,f). At two depth ranges, energetic shear energy generally corresponds to large forcing curl, mainly located in the reference SLA ensemble. In comparing with shear energy between two depth ranges, the significant change by a factor of 2 can be found in positive and negative SLA ensembles. However, their change trends are opposite, corresponding to the increase in positive SLA ensemble and the decrease in negative SLA ensemble, respectively.

The results obtained from ensemble and scatter analyses are consistent with those inferred from scenario studies. The critical values to separate different ensembles are selected at the point where evident changes of shear energy occur, clearly shown in scatter plots. However, these values cannot be regarded as the universal boundary to define different ensembles. We still need more samples to obtain toward-unified critical value, although there are numerous profiles in our 3-year observation.

Previous studies (e.g., Alford *et al*.^2^ and Jing *et al*.^29^) have revealed seasonal variations of near-inertial energy and its inducing mixing. Here, a 3-year long mooring observation provides us a good opportunity to examine the variability of near-inertial shears. Time series of mean shear energy over 80–800 m are shown in Fig. 4i,j. Unlike previous studies^2,29^, variation of downward shear did not strictly follow a seasonal cycle of wind stress (thick line in Fig. 4c,d), and was strongly influenced by cases of large curls and interannual change of SLA. Most of the large shear occurred in the wake of strong and moderate curls. From September 2015 to May 2016, negative SLA limited the mean shear energy to low values. These features revealed from time variation further support our abovementioned findings.

In summary, the influencing factors on intensity and propagation depth of near-inertial downward shear energy are discussed and some conclusions can be reached. 1) The response of shear intensity below the pycnocline to the increasing curl is non-monotone. Because very strong curl can stall the downward propagation of strong shear through decreasing internal wave flux below the pycnocline. Hence, it is the moderate (even weak) cyclone rather than the very strong cyclone that makes more contributions to the sub-pycnocline mixing. 2) The large positive and negative SLA fields cause the accumulation of large shear in the lower and upper parts of the pycnocline through inducing downwelling and upwelling motions, respectively. These conclusions give us some hints on how to improve the capability of simulating wind-induced near-inertial energy. Inaccurate and unmatched fields of wind stress curl and SLA, and ignorance of important contributions from the moderate and weak curls will probably misestimate the role of wind-induced near-inertial waves on the pycnocline and sub-pycnocline mixing.

In this study, observed velocity profiles spanning over 80–800 m lends us the opportunity to study the responses of near-inertial shears within and below the pycnocline to different forcings. During the whole observation period, intensities of all cyclones were not strong enough to reach the criteria of typhoon; however, this instead provides us an opportunity to study how the near-inertial shears respond to common cyclones, which are widely existing but drawn fewer attentions before. Another good opportunity for this study is that the corresponding SLA fields can be nearly equally divided into positive and negative periods, and amplitudes of SLAs during two periods were almost the same. This boosts the accuracy in estimating the influence of SLA on near-inertial shear. Although some influencing factors on intensity and propagation of near-inertial shear are discussed, it is not possible here to draw the conclusion of all forcings, such as the parametric subharmonic interaction (PSI), nonlinear wave-wave interaction, and moving speed and direction of cyclone. Nevertheless, we still hope that this study can deepen our knowledge of controlling mechanisms of downward near-inertial shear, and thus improve the capability of simulating NIWs (Fig. 3e,f). In the future, the structure, variability, and mechanism of upward energy propagating shear and their relationships with downward shear will be considered.

## Methods

### Mooring measurement and data processing

The subsurface mooring was located at 139.1°E, 11.6°N with a water depth of ~5200 m. This mooring is a part of the Scientific Observing Network of the Chinese Academy of Sciences (CASSON) in the western Pacific Ocean. The mooring was initially deployed on 14 August 2014 and recovered on 2 December 2017. On 7 November 2015 and 18 November 2016, the mooring was redeployed twice for maintenance. One upward- and one downward-looking TRDI 75 kHz ADCPs were equipped on the main float of the mooring at ~450 m. Two ADCPs measured velocity profiles over 50–1000 m with a bin size of 8 m every one hour. Data were then vertically interpolated into standard depth. Record gaps during the mooring redeployment were linearly interpolated.

In order to eliminate the change of internal waves’ amplitude and vertical wavelength due to variations in the buoyancy frequency as internal waves propagate vertically, zonal and meridional velocities (*u* and *v*) were WKB scaled^31^ as

1 $$u(z)=u\sqrt{{N}_{0}/\overline{N(z)}},\,v(z)=v\sqrt{{N}_{0}/\overline{N(z)}},$$

where $\overline{N(z)}$ is climatological buoyancy frequency profile computed from WOA13 temperature and salinity, and *N*_0_ is a reference buoyancy frequency calculated from the depth mean of $\overline{N(z)}$ over 50–1000 m. Near-inertial velocity was obtained by applying a first-order Butterworth band-pass filter over (1 ± 0.3) *f* to original velocity time series. Vertical shears of zonal and meridional velocities (*Su* = (∂*u*/∂*z*) and *Sv* = (∂*u*/∂*z*)) were calculated by first-differencing velocities over 8 m interval.

The wavenumber-frequency spectrum of velocity or shear is given as

2 $$\phi (\omega ,m)=\frac{|\tilde{w}\,\times {\tilde{w}}^{\ast }|}{max|\tilde{w}\,\times {\tilde{w}}^{\ast }|},$$

where *w* is complex velocity (*w(t, z)* = *u(t, z)* + *iv(t, z)*) or shear (*w(t, z)* = S*u(t, z)* + *iSv(t, z)*), w^*^ is the complex conjugate of *w*, which has been demeaned and detrended, $\tilde{w}$ is 2D Fourier transform of w, and $max|\tilde{w}\,\times {\tilde{w}}^{\ast }|$ denote the maximum of $|\tilde{w}\,\times {\tilde{w}}^{\ast }|$ over time and depth^9^. In frequency-wavenumber spectrum, positive and negative frequencies correspond to counterclockwise and clockwise rotations, respectively, and positive and negative wavenumbers indicate upward and downward energy propagations, respectively. The total velocity and shear fields can be decomposed into upward and downward propagating constituents through 2D Fourier filtering^9,31^.

### Internal wave energy fluxes

Internal wave energy flux (*F*_*E*_) can be used to identify the vertical propagation of energy density (*E*), and can be written as

3 $${F}_{E}=\langle w^{\prime} p^{\prime} \rangle ={C}_{gz}E,$$

where $p^{\prime}$ and $w^{\prime}$ are internal wave–induced perturbations in pressure and vertical velocity, < … > is phase average, and *C*_*gz*_ is the vertical group velocity. The detailed internal wave energy equation can be referred to Nash *et al*.^26^.

For the second term of Eq. 3, the pressure anomaly $p^{\prime} (z,t)$ is calculated from the density anomaly using the hydrostatic equation

4 $$p^{\prime} (z,t)={p}_{surf}(t)+{\int }_{z}^{0}\rho ^{\prime} (\hat{z},t)gd\hat{z},$$

where ${p}_{surf}(t)$ is the surface pressure, $\rho ^{\prime} (z,t)$ is the density perturbation, and can be estimated in terms of the vertical displacement of an isopycnal *ξ(z, t)* relative to its time mean position, that is

5 $$\rho ^{\prime} (z,t)=(\bar{\rho }/g)\overline{{N}^{2}}\xi (z,t),$$

where $\bar{\rho }$ is the time mean vertical density profile, *N* is stratification, and *g* is the gravitational acceleration.

For the third term of Eq. 3, *C*_*gz*_ needs to be deduced from the dispersion relation of NIWs^2^. The dispersion relation of NIWs is

6 $${\omega }^{2}={f}^{2}+{N}^{2}\frac{({k}^{2}+{l}^{2})}{{m}^{2}}={f}^{2}+{N}^{2}\frac{({k}_{H}^{2})}{{m}^{2}},$$

where *ω* is the wave frequency, (*k, l, m*) is a three-dimensional wave vector, *K*_*H*_ = *k*^2^ + *l*^2^ and *m* are horizontal and vertical wavenumbers, respectively. The vertical component of group speed for frequencies close to *f* is

7 $${c}_{gz}=\frac{\partial \omega }{\partial m}\approx \frac{-{N}^{2}{k}_{H}^{2}}{{m}^{3}f}.$$

Therefore, internal wave energy flux is closely related to stratification.

### Data sources

Monthly variation of stratification profiles during the mooring measurement period is calculated from EN4.2.1 temperature and salinity, which are downloaded from https://www.metoffice.gov.uk/hadobs/en4/. Climatological temperature and salinity are obtained from WOA13 dataset (https://www.nodc.noaa.gov/OC5/woa13/woa13data.html). Daily wind stress data are obtained from Advanced SCATterometer (ASCAT) global wind dataset (http://apdrc.soest.hawaii.edu/las/v6/dataset?catitem=12490). Daily sea level anomaly (SLA) data are obtained from Archiving Validation and Interpretation of Satellite Oceanographic Data (AVISO) gridded dataset (https://www.aviso.altimetry.fr/en/home.html). Values of temperature, salinity, wind stress, and SLA at the mooring site are derived through calculating the average over 138.6–139.6°E and 11.1–12.1°N. Modeled daily velocity from HYbrid Coordinate Ocean Model (HYCOM) are downloaded from http://tds.hycom.org/thredds/dodsC/GLBu0.08/expt_91.2/.
